# O-29 USING PRISONER MOVEMENT DATA TO IDENTIFY AND SCREEN CONTACTS OF A CASE OF MULTIDRUG RESISTANT TUBERCULOSIS (MDR-TB) IN A PRISON IN YORKSHIRE AND HUMBER

**DOI:** 10.1093/pubmed/fdag046.025

**Published:** 2026-07-09

**Authors:** Mrs Eliza Thompson-Westmacott, Lara Utsi, Helen Bagnall, Dr Nicholas Aigbogun, Christus Ferneyhough, Michael Kettles, Dr Patrick Lillie, Joanne Robinson, Andy Tomlinson, Jade Horn

**Affiliations:** UK Health Security Agency (UKHSA); UK Health Security Agency (UKHSA); UK Health Security Agency (UKHSA); UK Health Security Agency (UKHSA); UK Health Security Agency (UKHSA); UK Health Security Agency (UKHSA); Hull University Teaching Hospitals Trust; Integrated Community Services, City Health Partnership CIC; HMP Hull; Spectrum Community Health CIC

## Abstract

**Background:**

The Health Protection Team (HPT) were notified of an individual with infectious multi-drug resistant Tuberculosis (MDR-TB) in a prison. This was the first reported case in the UK secure estate of the AA041-2 strain which has caused outbreaks in prisons globally and is regarded as highly transmissible.

**Objectives:**

To identify, screen, test and treat contacts of the case and prevent onward transmission.

**Methods:**

Contacts were classified as prisoners or staff present in the prison while the individual was infectious (for approximately 7 weeks). Inmate movement data was extracted by His Majesty’s Prison and Probation Service (HMPPS) and summarised by Field Services to identify contacts. Interferon-gamma release assay (IGRA) screening at two prison sites was carried out by local TB services.

**Results:**

1,503 potential contacts were identified, including 21 staff, 467 inmates still in the prison at the time of the first screening and 1,015 inmates who had been transferred or released. 732 contacts (49%) who shared a wing with the case were prioritised for screening, including 467 inmates still in prison, 244 transferred contacts and 21 staff. 319 contacts (44%) were screened. 19 individuals were positive on IGRA (6%) including the cellmate of the index case, seventeen prisoner contacts and one staff member. No cases of active TB infection were identified.

**Conclusions:**

Despite requiring substantial resources this multi-agency response promptly collected and collated information and conducted mass screening for a large number of contacts in this high turnover setting to prevent further cases of MDR-TB. 6% positivity is in line with that expected in the UK prison population suggesting no elevated transmission, and no further active infection was identified in those screened, therefore no further screening was carried out. Improving data sharing methods would simplify collaborative working practices and improve the speed of response.

